# Supplementary material for: Temperature measurement of Quark-Gluon plasma at different stages
Source: Nat Commun. 2025 Oct 14;16:9098. doi: 10.1038/s41467-025-63216-5 (PMC12521530; doi:10.1038/s41467-025-63216-5)
Supplement: Supplementary file 1 — Supplementary Information [file 41467_2025_63216_MOESM1_ESM.pdf]

Supplementary Information

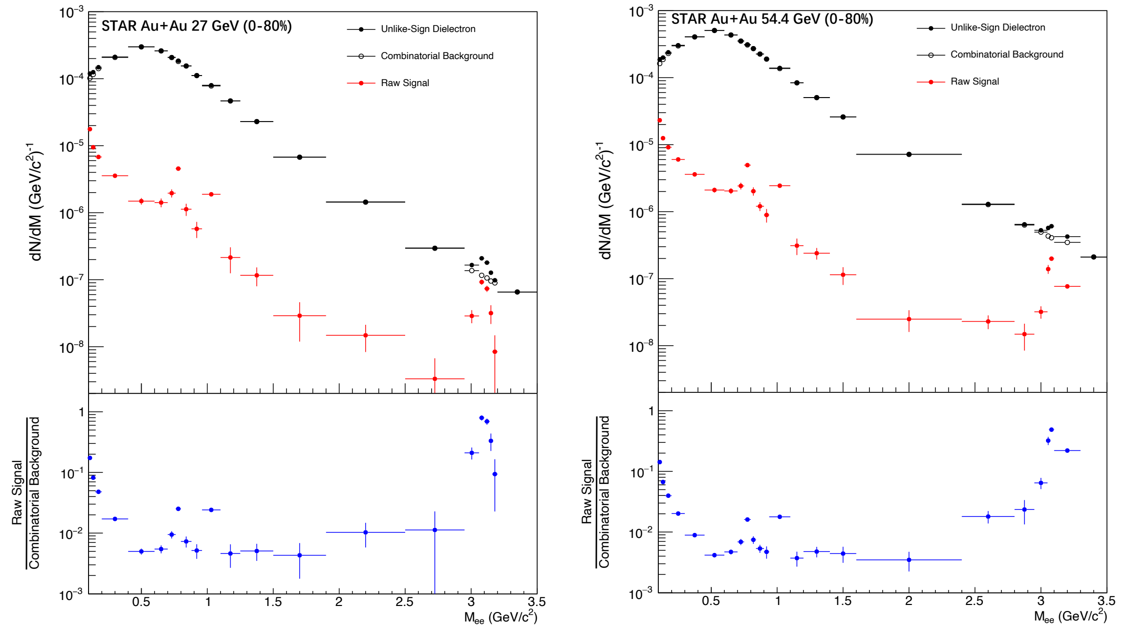

Supplementary Fig. 1: Raw signal and signal over background ratio of $\sqrt{s_{\mathrm{NN}}} = 27$ (left) and 54.4 GeV(right) Au+Au 0-80% centrality collisions.The vertical bars around data points represent the statistical uncertainties.

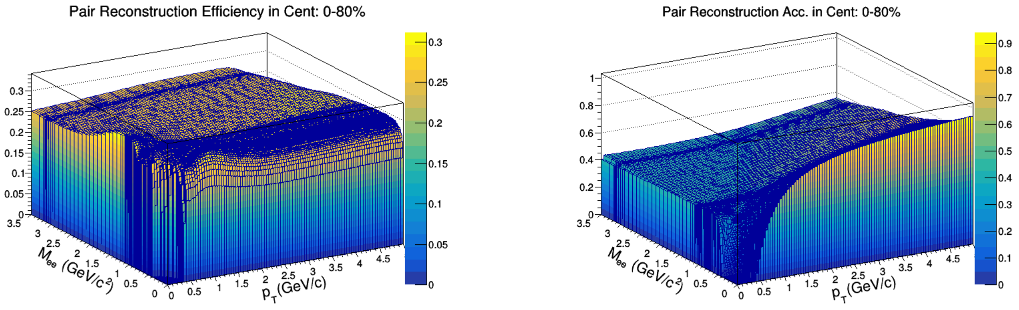

Supplementary Fig. 2: Example of dielectron pair reconstruction efficiency (left) and acceptance (right) in 2D (M_{ee}, p_{T}) of $\sqrt{s_{\mathrm{NN}}} = 27$ GeV Au+Au 0-80% centrality collisions

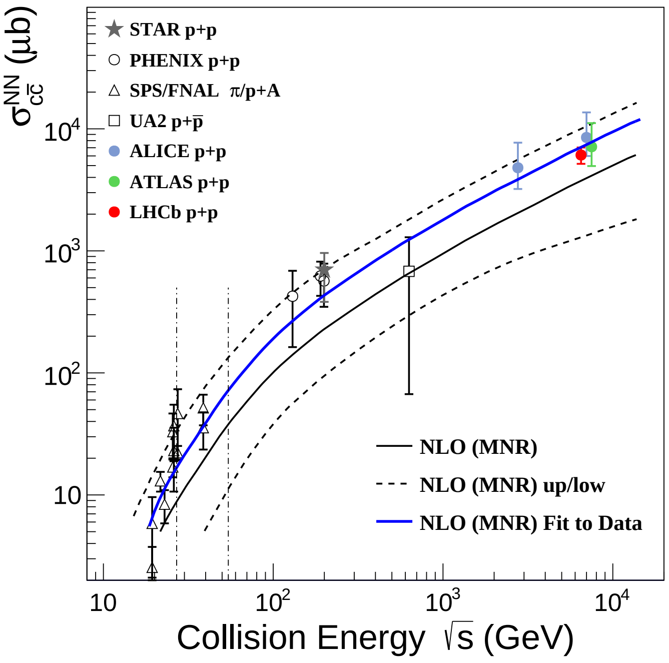

Supplementary Fig. 3: Total charm ($c\bar{c}$) production cross section as a function of collision energy. Data points are from world-wide experimental measurements [1–7]. Theoretical calculations of next-to-leading-order (NLO) pQCD (MNR [8]) and their upper/lower limits are shown as black solid and dashed curves. The blue curve shows the result using the MNR line shape to fit world-wide data. Vertical bars around data points represent the total experimental uncertainties.

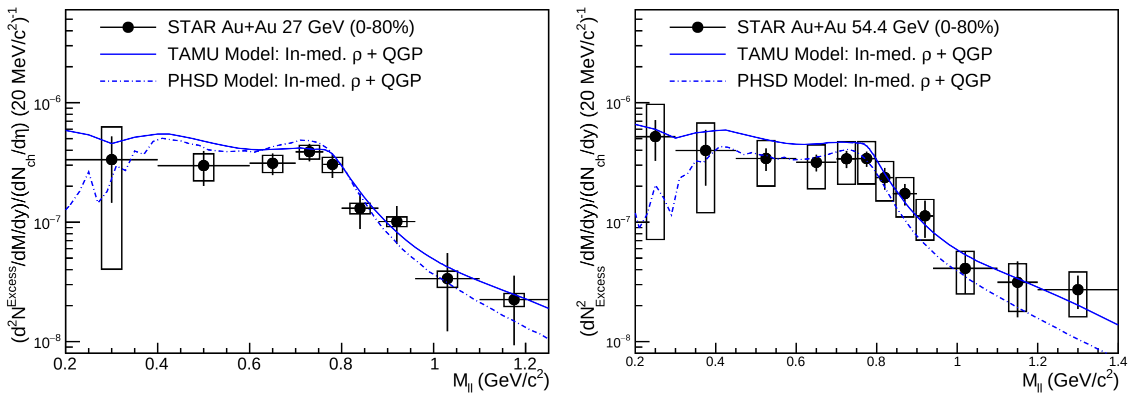

Supplementary Fig. 4: Low mass excess dielectron invariant mass spectrum for Au+Au collisions at $\sqrt{s_{\rm NN}} = 27$ GeV (left) and 54.4 GeV (right), normalized by $dN_{\rm ch}/dy$, compared to the theoretical calculations from the TAMU [9–12] and PHSD [13,14] models. Vertical bars and boxes around data points represent the statistical and systematic uncertainties, respectively.

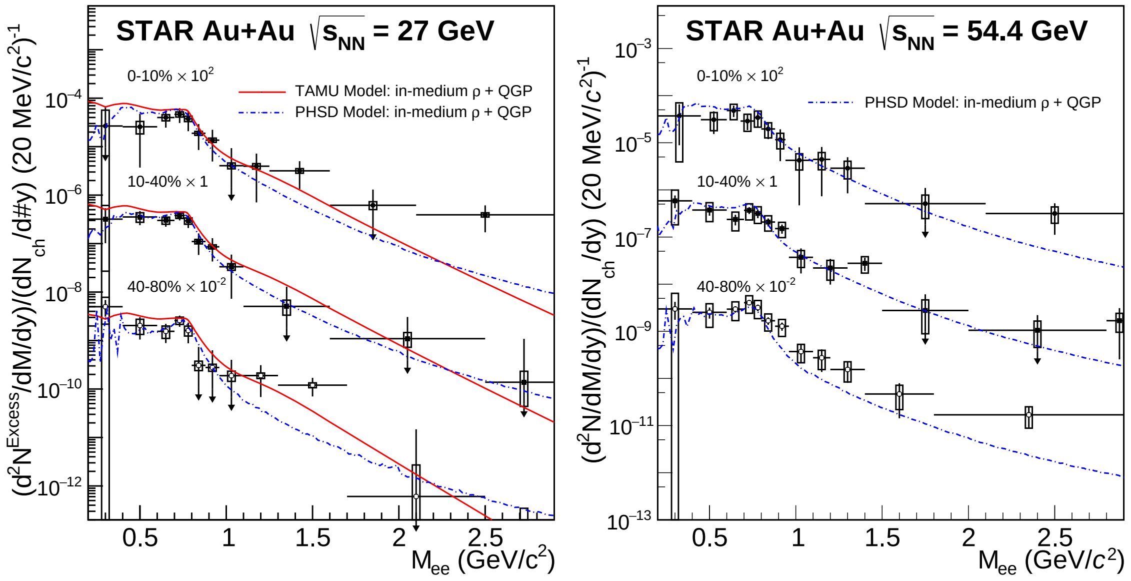

Supplementary Fig. 5: Excess dielectron invariant mass spectrum for Au+Au collisions at $\sqrt{s_{\mathrm{NN}}} =$ 27 GeV (left) and 54.4 GeV (right) in different centralities, compared to the theoretical calculations from the TAMU model (omitted in 54.4 GeV) [9–12] and the PHSD [13,14] model. Vertical bars and boxes around data points represent the statistical and systematic uncertainties, respectively.

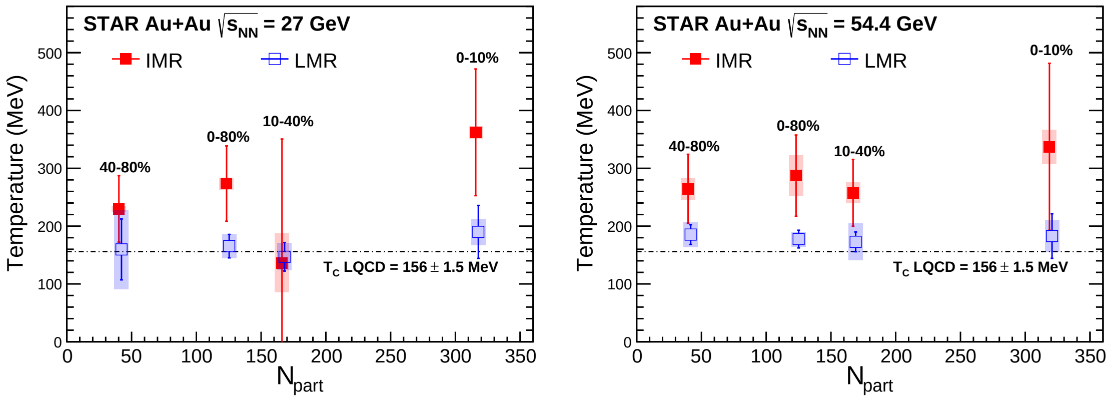

Supplementary Fig. 6: Temperatures extracted from LMR and IMR as a function of the number of participating nucleons ($N_{\rm part}$) of $\sqrt{s_{\rm NN}} = 27$ GeV(left) and 54.4 GeV(right) Au+Au collisions. The dot-dash line shows the pseudo-critical temperature derived in LQCD calculations evaluated at $\mu_{\rm B} = 0$. Vertical bars and shaded boxes around data points represent the statistical and systematic uncertainties, respectively.

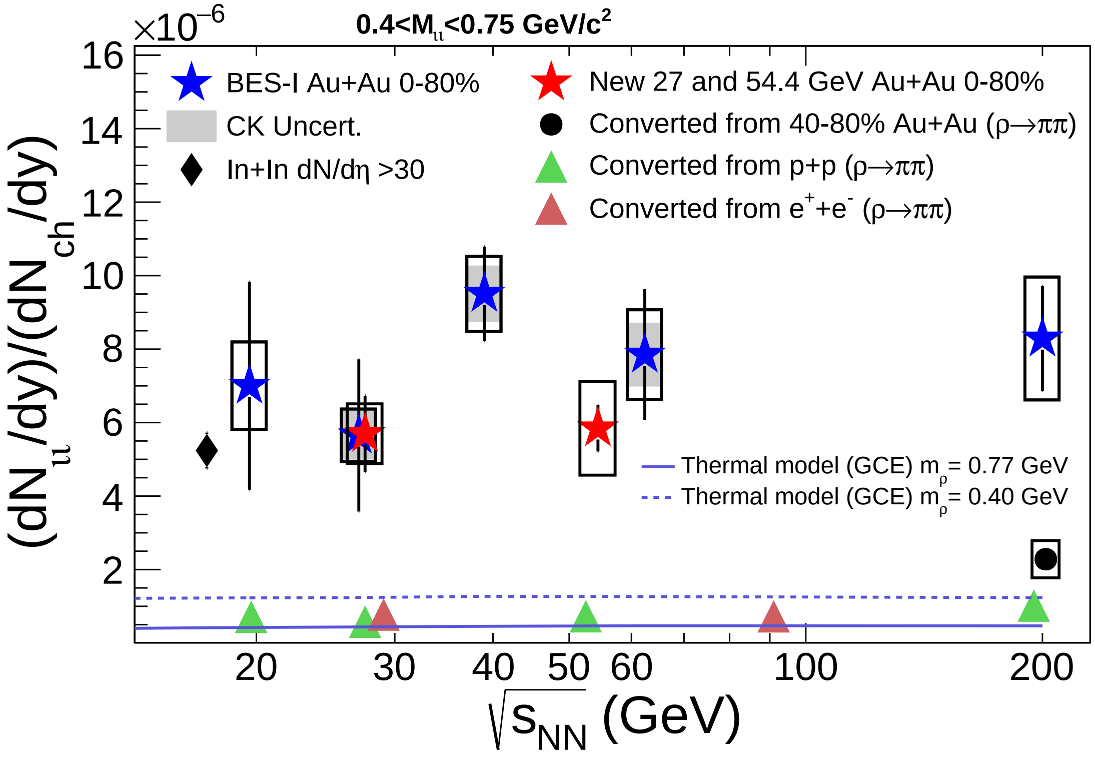

Supplementary Fig. 7: The collision-energy dependence of the integrated thermal dilepton yields in the mass range $0.4 < M_{ll} < 0.75$ GeV/c^2, normalized by the charged particle density $dN_{\rm ch}/dy$. Experimental data from this study and STAR BES-I data [15,16] are shown as red and blue stars, respectively. Triangles and the black circle represent the expected $\rho \to e^+e^-$ result based on the $\rho \to \pi^+\pi^-$ data measured in p+p collisions [17–20], $e^+ + e^-$ collisions [21–23] and Au+Au peripheral (40-80% centrality) collisions [24]. Vertical bars and open boxes around data points represent the statistical and systematic uncertainties, respectively. The solid and dashed lines show the theoretical prediction from the statistical thermal model (GCE) with chemical freeze-out parameters obtained by fitting to STAR measured hadrons [25,26].

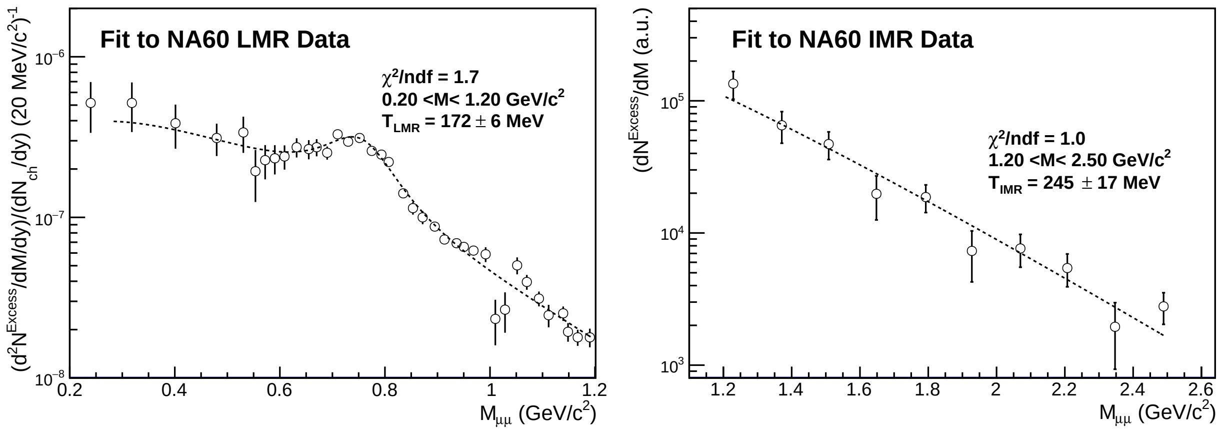

Supplementary Fig. 8: The left panel shows temperature extraction from the NA60 published LMR thermal dimuon spectra [27,28]. The right panel shows temperature extraction from the NA60 published IMR thermal dimuon spectra [29]. Vertical bars in the left panel represent statistical uncertainty while vertical bars in the right panel denote the sum in quadrature of statistical and systematic uncertainties.

Supplementary References

1. Abelev, B. *et al.* Measurement of charm production at central rapidity in proton-proton collisions at $\sqrt{s} = 7$ TeV. *JHEP* **01**, 128 (2012). 1111.1553.

2. Aaij, R. *et al.* Prompt charm production in pp collisions at $\sqrt{s} = 7$ TeV. *Nucl. Phys. B* **871**, 1–20 (2013). 1302.2864.

3. Adamczyk, L. *et al.* Measurements of D^0 and D^* Production in $p + p$ Collisions at $\sqrt{s} = 200$ GeV. *Phys. Rev. D* **86**, 072013 (2012). 1204.4244.

4. Adare, A. *et al.* Heavy Quark Production in $p + p$ and Energy Loss and Flow of Heavy Quarks in Au+Au Collisions at $\sqrt{s_{\rm NN}} = 200$ GeV. *Phys. Rev. C* **84**, 044905 (2011). 1005.1627.

5. Aad, G. *et al.* Measurement of $D^{*\pm}$, $D^{\pm}$ and $D_s^{\pm}$ meson production cross sections in pp collisions at $\sqrt{s} = 7$ TeV with the ATLAS detector. *Nucl. Phys. B* **907**, 717–763 (2016). 1512.02913.

6. Alves, G. A. *et al.* Forward cross-sections for production of D^+, D^0, D_s, D^{*+} and Λ^c in 250 GeV $\pi^{\pm}$, $K^{\pm}$, and p nucleon interactions. *Phys. Rev. Lett.* **77**, 2388–2391 (1996). [Erratum: Phys.Rev.Lett. 81, 1537 (1998)].

7. Amaryan, M. *et al.* (eds.). *Workshop on Excited Hyperons in QCD Thermodynamics at Freeze-Out (YSTAR2016) Mini-Proceedings* (2017). 1701.07346.

8. Mangano, M. L., Nason, P. & Ridolfi, G. Heavy quark correlations in hadron collisions at next-to-leading order. *Nucl. Phys. B* **373**, 295–345 (1992).

9. van Hees, H. & Rapp, R. Comprehensive interpretation of thermal dileptons at the SPS. *Phys. Rev. Lett.* **97**, 102301 (2006). `hep-ph/0603084`.

10. Rapp, R. Signatures of thermal dilepton radiation at RHIC. *Phys. Rev. C* **63**, 054907 (2001). `hep-ph/0010101`.

11. Rapp, R. & van Hees, H. Thermal Dileptons as Fireball Thermometer and Chronometer. *Phys. Lett. B* **753**, 586–590 (2016). `1411.4612`.

12. Rapp, R. & van Hees, H. Thermal Electromagnetic Radiation in Heavy-Ion Collisions. *Eur. Phys. J. A* **52**, 257 (2016). `1608.05279`.

13. Cassing, W., Bratkovskaya, E. L., Rapp, R. & Wambach, J. Probing the ρ^0 spectral function in hot and dense nuclear matter by dileptons. *Phys. Rev. C* **57**, 916–921 (1998). `nucl-th/9708020`.

14. Cassing, W. & Bratkovskaya, E. L. Hadronic and electromagnetic probes of hot and dense nuclear matter. *Phys. Rept.* **308**, 65–233 (1999).

15. Adamczyk, L. *et al.* Energy dependence of acceptance-corrected dielectron excess mass spectrum at mid-rapidity in Au+Au collisions at $\sqrt{s_{\mathrm{NN}}} = 19.6$ and 200 GeV. *Phys. Lett. B* **750**, 64–71 (2015). `1501.05341`.

16. Abdulhamid, M. I. *et al.* Measurements of dielectron production in Au+Au collisions at $\sqrt{s_{\mathrm{NN}}} = 27$, 39, and 62.4 GeV from the STAR experiment. *Phys. Rev. C* **107**, L061901 (2023).

17. Blobel, V. *et al.* Observation of Vector Meson Production in Inclusive p+p Reactions. *Phys. Lett. B* **48**, 73–76 (1974).

18. Singer, R. *et al.* ρ^0 Production in 205 GeV/c p p Interactions. *Phys. Lett. B* **60**, 385–388 (1976).

19. Aguilar-Benitez, M. *et al.* Inclusive particle production in 400 GeV p+p interactions. *Z. Phys. C* **50**, 405–426 (1991).

20. Drijard, D. *et al.* Production of Vector and Tensor Mesons in Proton Proton Collisions at $\sqrt{s} = 52.5$ GeV. *Z. Phys. C* **9**, 293 (1981).

21. Derrick, M. *et al.* Neutral K^* (890) and ρ^0 Meson Production in e^+e^- Annihilation at the $\sqrt{s} = 29$ GeV. *Phys. Lett. B* **158**, 519–524 (1985).

22. Albrecht, H. *et al.* Inclusive production of $K^*(892)$, $\rho^0(770)$, and $\omega(783)$ mesons in the Υ energy region. *Z. Phys. C* **61**, 1–18 (1994).

23. Pei, Y.-J. A simple approach to describe hadron production rates in e^+e^- annihilation. *Z. Phys. C* **72**, 39–46 (1996).

24. Adams, J. *et al.* ρ^0 production and possible modification in Au+Au and p+p collisions at $\sqrt{s_{\mathrm{NN}}}$ = 200 GeV. *Phys. Rev. Lett.* **92**, 092301 (2004). `nucl-ex/0307023`.

25. Wheaton, S. & Cleymans, J. THERMUS: A Thermal model package for ROOT. *Comput. Phys. Commun.* **180**, 84–106 (2009). `hep-ph/0407174`.

26. Adamczyk, L. *et al.* Bulk Properties of the Medium Produced in Relativistic Heavy-Ion Collisions from the Beam Energy Scan Program. *Phys. Rev. C* **96**, 044904 (2017). 1701.07065.

27. Arnaldi, R. *et al.* Evidence for radial flow of thermal dileptons in high-energy nuclear collisions. *Phys. Rev. Lett.* **100**, 022302 (2008). 0711.1816.

28. Arnaldi, R. *et al.* NA60 results on thermal dimuons. *Eur. Phys. J. C* **61**, 711–720 (2009). 0812.3053.

29. Arnaldi, R. *et al.* Evidence for the production of thermal-like muon pairs with masses above 1 GeV/c^2 in 158 A GeV Indium-Indium Collisions. *Eur. Phys. J. C* **59**, 607–623 (2009). 0810.3204.
